# The relationship among social capital, eHealth literacy and health behaviours in Chinese elderly people: a cross-sectional study

**DOI:** 10.1186/s12889-020-10037-4

**Published:** 2021-01-06

**Authors:** Guang-Hui Cui, Shao-Jie Li, Yong-Tian Yin, Li-Jun Chen, Jia-Qin Li, Feng-Yuan Liang, Xin-Yao Liu, Lei Chen

**Affiliations:** 1 School of Acupuncture and Tuina, Shandong University of Traditional Chinese Medicine, Jinan, 250355 China; 2 Department of Social Medicine and Health Service Management, Xiangya School of Public Health, Central South University, Changsha, 410078 China; 3 School of Nursing, Shandong University of Traditional Chinese Medicine, Jinan, 250355 China; 4 School of Ophthalmology and Optometry, Shandong University of Traditional Chinese Medicine, Jinan, 250355 China; 5 School of Chinese Medicine, Shandong University of Traditional Chinese Medicine, Jinan, 250355 China

**Keywords:** Social capital, eHealth literacy, Health behaviours, Elderly people

## Abstract

**Background:**

Social capital has been linked to health behaviours, but the underlying mechanism is unclear. Previous studies have found that health literacy played the role of a mediator in the relationships among social capital, individual physical activity and nutrition. But it is not clear whether eHealth literacy mediates the impact of social capital on health behaviours. Therefore, our research aimed to explore the relationships among social capital (structural and cognitive social capital), eHealth literacy, and the health behaviours of elderly people, and to analyse the mediating effect of eHealth literacy, while providing a theoretical basis for a health behaviour intervention for elderly people.

**Methods:**

From January to February 2019, we conducted a cross-sectional survey of 1201 Chinese people aged over 60 years using the Chinese Shortened Social Capital Scale (contains two subscales of structural social capital and cognitive social capital), eHealth Literacy Scale, and Health-Promoting Lifestyle Profile. We used structural equation modelling to test a hypothetical mediation model.

**Results:**

The mean scores of social capital was 72.07 (SD = 13.03), 17.24 (SD = 9.34) for eHealth literacy, and 112.23 (SD = 23.25) for health behaviours. Social capital and eHealth literacy were significantly correlated with health behaviours, and social capital and structural social capital were significantly correlated with eHealth literacy. Lastly, eHealth literacy mediated the relationship between structural social capital and health behaviours.

**Conclusions:**

eHealth literacy was an important mediating factor for elderly people’s structural social capital and health behaviours. Therefore, social capital and eHealth literacy must be considered when designing and implementing health behaviour intervention programmes for elderly people.

## Introduction

According to the data of the National Bureau of Statistics of China, there were 249 million people aged 60 years and over in China by the end of 2018, which accounted for 17.9% of the total population [1]. With the continued growth of the ageing population, there has been increasing demand for the medical and health management of elderly people [2]. Research shows that about two-thirds of elderly people over 65 years old suffer from two or more chronic diseases at the same time [3], which leads to a significant increase in the use of public health resources and in medical expenditure [4]. In the context of relatively insufficient medical resources, research into health promotion and disease prevention that uses the perspective of social capital has gradually become a new direction in the field of public health [5]. Social capital refers to the social structural resources owned by individuals, which are embodied in trust, norms, and networks [6] . In order to understand and analyse the connotation of social capital and its role in individual life and measure it further, Uphoff considered that it can be divided into structural and cognitive types [7]. In health research, this distinction has been one of the most influential viewpoints and gained currency [8, 9]. The structural social capital refers to the precedent, rules and behaviour norms formed by human participation in social life, as well as the role and social network determined by social interaction activities [7]. The cognitive social capital refers to people’s spiritual experience such as trust, reciprocity, sense of belonging, and subjective will such as attitude, sharing and values, which can be simply considered as what people ‘perceive’ in social relationships [10]. At present, many studies have explored the relationship between social capital and individual health. For example, it has been shown that social capital was positively related to the self-rated health status of elderly people [11]. A systematic review reconfirmed the view that social capital was a protective factor for the individual’s health [12]. Furthermore, poor social capital may be related to the mortality rate of elderly people due to chronic diseases and suicide [13]. Therefore, it is of great public health significance to study the influence of social capital on health of the elderly people.

From the perspective of the modern medical model, the maintenance of health and emergence of diseases can be affected by multiple remote and proximal factors. According to the health-related risk factor model [14], social structure and the social resources that it includes are remote factors that affect individual health, and its mechanism must include proximal mediating behavioural factors. For example, a previous study has shown that lifestyle can mediate the relationship between social capital and health [15]. Regarding the proximal factors, health-promoting lifestyles, which refers to a kind of spontaneous and multi-level health behaviour and perception that is produced by individuals to maintain or improve their health status [16], includes exercise, nutrition, health responsibility, interpersonal support, self-actualisation, and stress management [17]. Among these, the health-related lifestyle and behaviour (the proximal factor), which includes smoking, diet, and exercise, has been proved to be the most direct and effective factor for intervening in people’s health [18]. Researchers have reported that social capital is positively correlated with fruit and vegetable intake among rural adults [19]. In addition, social capital and social participation are related to physical inactivity [20], smoking [21], and insufficient sleep [22]. A cross-sectional survey of elderly people in Iran demonstrated that there was a positive correlation between health behaviours and social capital [2]. Although some studies have found that social capital is closely related to lifestyle, there is still a lack of research into the mechanism underlying social capital and health behaviours.

Health literacy is also considered to be an important proximal factor that affects health [23]. Nutbeam believes that health literacy has three levels: functional literacy, communicative literacy, and critical literacy [24]. The first two levels emphasise the acquisition and dissemination of health information by individuals. Waverijn indicated that social capital can speed up the circulation of existing health information in social networks and continuously increase the amount of new information resources [25]. This shows that there is a correlation between health literacy and social capital [26]. In addition, a study from China found that health literacy played the role of a mediator in the relationships among social capital, individual physical activity and nutrition [27]. However, it is not clear whether it generalises to eHealth literacy.

eHealth literacy is defined as the individual’s ability to search for, understand, and estimate health information using electronic media devices and to use the obtained information to process and solve personal health problems [28]. The extension and renewal of eHealth literacy as a concept of health literacy may also be related to individual social capital, but this has rarely been reported in the literature. Previous studies have shown that the eHealth literacy of college students [29], nurses [30], and patients with Type 2 diabetes [31] was positively correlated with health behaviours. However, the relationship between the two factors has not been fully confirmed among elderly people. In recent years, Internet technology, which has developed rapidly, has become an important source of people’s health-related information. The use and development of online health information could help to meet the increasing health demands of elderly people, especially in remote rural areas [32]. This means that improving elderly people’s ability to use this source of information could be an effective way to alleviate the shortage of health resources and to improve the accessibility of health services. Therefore, it is of great public health significance to inquire into the relationship between individual social capital and eHealth literacy.

**Fig. 1:**
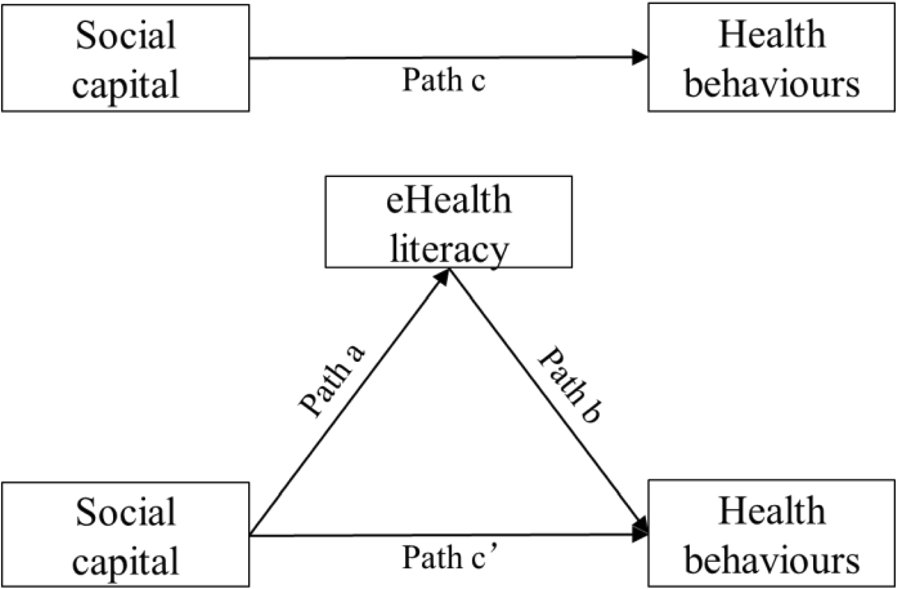
The hypothesised model

In summary, social capital and eHealth literacy are closely related to health behaviours. It is important to clarify the relationship between these three factors in order to explain the positive role of social capital at the level of individual health and to improve elderly people’s quality of life. Hsu et al. found that eHealth literacy plays an intermediary role between personal factors and health behaviours [33]. From this, we can infer that eHealth literacy may have an intermediary effect on the association between elderly people’s social capital and health behaviours (Fig. 1). Based on our cross-sectional study of elderly people in China, which is the first to examine the relationship between their social capital, eHealth literacy, and health behaviours, we proposed the following hypotheses:

- Hypothesis 1: Social capital (structural and cognitive social capital) will be associated with elderly people’s eHealth literacy.
- Hypothesis 2: eHealth literacy will mediate the relationship between social capital (structural and cognitive social capital) and health behaviours.

## Materials and methods

### Participants

This cross-sectional study was based on data collected in Jinan City in January and February 2019. The sample size was estimated using the formula for epidemiologic study of means-continuous outcome: $\mathrm{n}=\frac{2{\left({Z}_{\alpha /2}+{Z}_{\beta}\right)}^2}{\delta^2}$[34]. In this study, we set the effect size δ of health behaviours to 0.15, the confidence level (CI) was 95%, and the power was 80%. Using Z_α/2_ = 1.96, Z_*β*_ = 0.84, and δ = 0.15, the calculated sample size was 697. Considering that there may be a loss to follow-up rate of 20%, the final estimated minimum sample size was 837. The participants were selected from four districts and two counties of Jinan City using stratified cluster random sampling. We selected two streets or towns from each district or county, and two urban communities or natural villages from each street or town. Finally, eight communities and 16 villages were selected for the investigation. The inclusion criteria were people 60 years of age or older, who were conscious and had normal language communication skills, and who had the ability to surf the Internet. We recruited medical college students as investigators, and they all received uniform training. Participants were carefully briefed about the study’s purpose and content. After obtaining their consent, the investigator helped them to complete the questionnaire. The study was conducted in accordance with the Declaration of Helsinki. Our research was ethically approved by the medical ethics committee of Central South University (identification code: CTXY-150002-7).

### Measures

Our instrument consisted of a questionnaire about the participants’ demographic information, and included social capital scale, eHealth literacy scale, and health-promoting lifestyle profile. The demographic information was obtained using a questionnaire that we produced. This included age, gender, education level, self-rated physical health, family finances, and marital status.

We employed the Chinese Shortened Social Capital Scale (CSSCS), which was designed by Xu et al. [35], to assess the participants’ social capital. The scale was developed based on the World Bank’s social capital assessment tool [36] and combined with China’s national conditions and culture. It has been widely used in the Chinese population [37–39]. It has two subscales: structural social capital and cognitive social capital. The structural social capital subscale includes three dimensions: social participation (four items), social support (four items), and social connection (three items). The cognitive social capital subscale consists of three dimensions: trust (three items), cohesion (five items), and reciprocity (three items). A self-assessed five-point Likert scale was used for each item. The responses ranged from ‘strongly inconsistent’ to ‘strongly consistent’ and they scored 1 to 5 points, respectively. The total score was 22–110 points. Higher scores indicate a higher level of social capital.

eHealth literacy was measured using the eHealth Literacy Scale (eHEALS) that was developed by Norman [28]. This is composed of eight items, and it mainly evaluates the individual’s ability to collect, evaluate, and apply online health information. A self-assessed five-point Likert scale was used for each item. The responses ranged from ‘strongly disagree’ to ‘strongly agree’ and they scored 1 to 5 points, respectively. The total score was 8–40 points. The scale has been translated into Chinese and it is the only eHealth literacy assessment tool used in the elderly people in China, and has good internal consistency and predictive validity [40, 41].

Health behaviours were measured using the Health-Promoting Lifestyle Profile (HPLP), which was developed by Walker et al. [17] and revised by Huang et al. [42]. The 42-item scale consists of six dimensions: self-actualisation (fourteen items), health responsibility (nine items), exercise (three items), nutrition (five items), interpersonal support (five items), and stress management (six items). A self-evaluated four-point Likert scale was used for each item. The responses ranged from ‘never’ to ‘always’ and they scored 1 to 4 points, respectively. The total score was 42–168 points. Higher scores indicate a greater amount of health behaviours. The scale was widely used to measure the health behaviours of the Chinese elderly people, because it contained more comprehensive multi-dimensional health behaviours, and it has good internal consistency (0.91), split-half reliability (0.92), retest reliability (0.68) and construct validity [43].

### Data analysis

The SPSS version 23 (IBM SPSS Statistics, Armonk, NY, USA) and AMOS version 24 software (IBM Corp., Armonk, NY, USA) were used to conduct the data analysis. All statistical tests were two-tailed and statistical significance for all analyses was set at 0.05. We first performed the normality test and homogeneity test of variance. Then, descriptive statistics were used to describe the participants’ sociodemographic characteristics and the CSSCS, eHEALS, and HPLP scores. Harman’s single-factor test was employed to assess common method bias [44]. Pearson’s *r* correlations were used to examine the associations between the CSSCS, eHEALS, and HPLP scores. The structural equation modelling (SEM) with maximum likelihood and bootstrapping methods were used to test the hypothesised mediation model (Fig. 1). Bootstrapping methods were set to 5000 samples and were used to test the 95% bias-corrected CI for indirect effect.

According to the principle of mediation analysis and relevant guidelines [45, 46], the mediation model test in this study should meet the following conditions: 1) The path from social capital (structural and cognitive social capital) to health behaviours (path c, Fig. 1) was significant; 2) The path from social capital (structural and cognitive social capital) to eHealth literacy (path a) was significant; 3) controlling for social capital (structural and cognitive social capital), the path from eHealth literacy to health behaviours (path b) was significant; and 4) the indirect effect of eHealth literacy (a*b) on the relationship between social capital and health behaviours was significant (the 95% CI did not include zero).

## Results

### Common method bias test

Harman’s single-factor test was used to assess the presence of common method bias. The results showed that the characteristic values of a total of 10 factors were greater than 1, and that the first common factor explained 29.527% of the total variation, which is less than the critical value of 50% [47]. This indicates that there was no serious common method bias in this study.

### Descriptive statistics

A total of 1250 questionnaires were distributed, and the final sample included 1201 elderly people (a response rate of 96.1%). The sample included 560 males (46.6%) and 641 females (53.4%). The participant group’s mean age was 70.12 years old (SD = 6.29) and 53.4% (*n* = 641) were female. Participants living in rural area accounted for 73.4% (*n* = 881). In terms of the participants’ education level, 59.3% (*n* = 712) was in primary school and below. The mean scores of social capital was 72.07 (SD = 13.03), 17.24 (SD = 9.34) for eHealth literacy, and 112.23 (SD = 23.25) for health behaviour. (Table 1).

**Table 1:** Descriptive statistics of the sample’s characteristics (*N* = 1201)

Characteristics	***N*** (%)	Mean	***SD***	Range
Age (years)		70.12	6.29	60.00–97.00
60–69	622 (51.8)			
70–79	485 (40.4)			
≥ 80	94 (7.8)			
Gender				
Male	560 (46.6)			
Female	641 (53.4)			
Residence				
Urban area	320 (26.6)			
Rural area	881 (73.4)			
Education level				
Primary school and below	712 (59.3)			
Junior middle school	331 (27.6)			
High school	124 (10.3)			
University/college and above	34 (2.8)			
Social capital		72.07	13.03	22.00–110.00
Structural social capital		30.81	6.67	11.00–55.00
Cognitive social capital		41.27	8.30	11.00–55.00
eHealth literacy		17.24	9.34	8.00–40.00
Health behaviours		112.23	23.25	42.00–168.00
Self-actualisation		38.88	8.29	14.00–56.00
Health responsibility		21.21	6.20	9.00–36.00
Exercise		7.15	2.48	3.00–12.00
Nutrition		15.22	2.72	5.00–20.00
Interpersonal support		13.87	3.23	5.00–20.00
Stress management		15.92	3.82	6.00–24.00

### Correlations between social capital, eHealth literacy, and health behaviours

The Pearson’s *r* correlations between social capital, eHealth literacy, and health behaviours are presented in Table 2. Social capital and eHealth literacy were significantly correlated with health behaviours, and social capital and structural social capital were significantly correlated with eHealth literacy.

**Table 2:** Correlations (*r*) between social capital, eHealth literacy, and health behaviours (*N* = 1201)

Variables	1	2	3	4	5	6	7	8	9	10	11
1. Social capital	1										
2. Structural social capital	0.84^**^	1									
3. Cognitive social capital	0.90^**^	0.51^**^	1								
4. eHealth literacy	0.21^**^	0.36^**^	0.05	1							
5. Health behaviours	0.51^**^	0.50^**^	0.40^**^	0.45^**^	1						
6. Self-actualisation	0.52^**^	0.46^**^	0.44^**^	0.38^**^	0.94^**^	1					
7. Health responsibility	0.38^**^	0.46^**^	0.22^**^	0.52^**^	0.87^**^	0.72^**^	1				
8. Exercise	0.33^**^	0.40^**^	0.20^**^	0.42^**^	0.78^**^	0.66^**^	0.70^**^	1			
9. Nutrition	0.47^**^	0.40^**^	0.42^**^	0.23^**^	0.76^**^	0.71^**^	0.55^**^	0.49^**^	1		
10. Interpersonal support	0.49^**^	0.42^**^	0.43^**^	0.30^**^	0.82^**^	0.76^**^	0.63^**^	0.55^**^	0.63^**^	1	
11. Stress management	0.43^**^	0.42^**^	0.33^**^	0.42^**^	0.90^**^	0.82^**^	0.74^**^	0.70^**^	0.62^**^	0.69^**^	1

Note: **p < 0.01

### Mediation model test

**Fig. 2:**
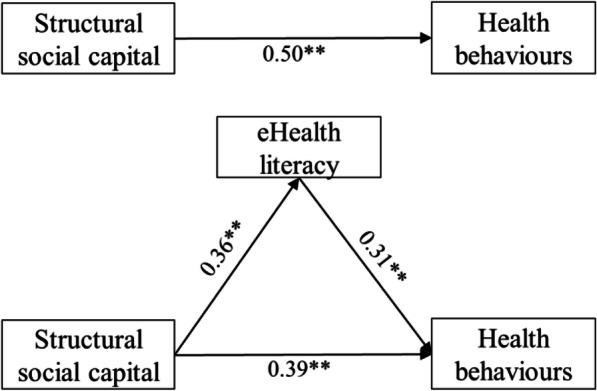
Model 1. Relationship between structural social capital and health behaviours, with eHealth literacy as a mediator. Note: **p < 0.01

The SEM with observed variables was used to test the association between social capital, eHealth literacy, and health behaviours. In Model 1, the paths from structural social capital to health behaviours (*β* = 0.39, *p* < 0.01) and to eHealth literacy (*β* = 0.36, *p* < 0.01) were significant (Fig. 2). The path from eHealth literacy to health behaviours (*β* = 0.31, *p* < 0.01) was also significant. The total effect of structural social capital on health behaviours was significant (*β* = 0.50, *p* < 0.01). Furthermore, the indirect effect of eHealth literacy on the relationship between structural social capital and health behaviours was significant (*β* = 0.11, *p* < 0.01). The bootstrap (5000 samples) standard error of 0.01 and bootstrap bias-corrected 95% CI (0.09, 0.14) did not include zero. Thus, we concluded that there was a significant mediation effect of eHealth literacy on the relationship between structural social capital and health behaviours.

**Fig. 3:**
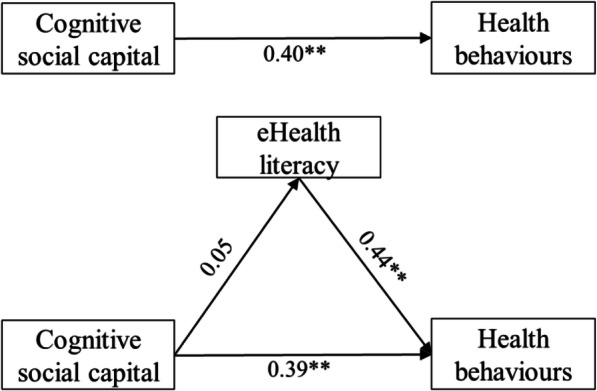
Model 2. Relationship between cognitive social capital and health behaviours, with eHealth literacy as a mediator. Note: **p < 0.01

In Model 2, the path from cognitive social capital to health behaviours (*β* = 0.39, *p* < 0.01) and the path from eHealth literacy to health behaviours (*β* = 0.44, *p* < 0.01) were significant (Fig. 3). However, the path from cognitive social capital to eHealth literacy (*β* = 0.05, *p* > 0.05) was not significant, which suggests that eHealth literacy does not mediate the relationship between cognitive social capital and health behaviours.

## Discussion

This cross-sectional survey systematically explored the relationship between the social capital, eHealth literacy, and health behaviours of elderly people in China. We found that there are significant positive correlations between social capital and structural social capital and eHealth literacy, which supports Hypothesis 1. Previous studies have also reported that online social capital is related to college students’ eHealth literacy [48]. Lee [49] also proposes that social capital is essentially a resource that is embedded in social networks, and that it has a positive effect on improving the efficiency of health information dissemination. To a certain extent, a high level of social capital provides elderly people with more opportunities for learning and communication [50], which is of great significance for them when mastering the use of electronic products, understanding the acquisition and evaluation of electronic health information, and improving eHealth literacy. Previous studies have also confirmed the positive effect of communicating with peers on the promotion of eHealth literacy [51]. In addition, Kim believes that social capital can be a source of individuals’ self-efficacy when finding, understanding, and using health information [52], which further shows that social capital may have a specific correlation with elders’ eHealth literacy.

This study’s results also indicated that the correlation between cognitive social capital and eHealth literacy was not statistically significant, which may be because cognitive social capital is biased towards individual perception. However, a previous study has shown that cognitive social capital does contribute to knowledge acquisition [53], which is inconsistent with our findings. Due to the lack of relevant evidence, we cannot infer why cognitive social capital is not related to eHealth literacy. Therefore, further verification is needed in the future.

There was a significant positive correlation between social capital and health behaviours, which is consistent with the results of studies of elderly people in Iran [2]. Research has shown that a low level of social capital is related to the physical activity of Brazilian adults and their inadequate fruit and vegetable intake, smoking, and other behaviours [54]. This indicates that social capital could play a role in correcting health-risk behaviours and increasing health-promoting behaviours. Based on previous research, the basic elements of social capital include networks, reciprocity norms, and trust [55, 56]. Among these, social objects, such as family, relatives, and friends in the network, can provide timely persuasion, supervision, and subtle influence, which help to strengthen elderly people’s sense of health responsibility and to motivate the maintenance of ideal health behaviours. For example, research from Mexico shows that encouragement from members of social networks is related to the motivation to eat more fruit and vegetables [57]. In addition, reciprocity norms are a common code of conduct and they act as a relationship model for people when they participate in social life, which is based on the network [56] and which has a collective behavioural orientation and restraint [2]. This behavioural orientation and restraint can help to encourage elderly people to follow the community’s or an organisation’s disease prevention and control and health management measures, and to actively understand and improve their own health behaviours.

The SEM showed that the elderly people’s eHealth literacy had an intermediary effect on the association between structural social capital and health behaviours. That is, structural social capital not only directly affected health behaviours but also had an indirect effect through eHealth literacy. This supports Hypothesis 2. As an important achievement of cognitive and behavioural science research in recent years, the theory of knowledge, attitude, and practice has been gradually applied in various health-related fields. The theory proposes that changes in human activities can be divided into the three uninterrupted stages of acquiring knowledge, generating beliefs, and forming behaviours [58]. On the one hand, social capital can directly provide health information and self-efficacy. In addition, the communication and interaction between members of the social network helps elderly people to understand and proficiently use electronic products and search engines. It can be seen that social capital provides elderly people with access to and support with knowledge, which plays an important role in improving eHealth literacy. On the other hand, when elderly people have a high level of eHealth literacy, their correct cognition and positive beliefs about health management and health promotion will be embedded in their health-related decision-making and used as a continuous motivation. It is beneficial for elderly people to use online health knowledge to regulate their own health behaviours. In addition, the Electronic Health Integrated Use Model indicates that people with better eHealth literacy are not only more inclined to use the Internet and electronic products to find answers to health-related questions but they also have a stronger belief in using this information to promote healthy behaviour [59].

This study suggests that the government should fully tap the potential of social capital and eHealth literacy when promoting elderly people’s health behaviours. In terms of social capital, it is necessary to explore the establishment of a new community management mechanism based on reciprocal cooperation, because it can create conditions for elderly people to participate in interpersonal communication and to increase their sense of trust and belonging. Thus, we suggest that the government should set up special fund projects to improve the construction of community facilities for the elderly people, popularize community activity rooms and universities for the elderly people, and provide a platform for enhancing the social network of the elderly people. At the same time, the community can establish a health management mutual assistance supervision group, organize health lectures and other activities, promote the exchange of health information and emotional exchanges, urge the elderly people to comply with reciprocal norms, and limit their own health-damaging behaviours. In terms of eHealth literacy, it is very important to reduce the digital divide, because previous studies have shown that the Internet use skills and frequency of the elderly people were significantly positively correlated with eHealth literacy [60]. We suggest the government can implement discounts or financial subsidies for elderly people to purchase electronic products. In addition, with the increase of age, the decline of vision, hearing and cognitive ability makes the elderly often suffer from technical difficulties [61]. Therefore, the development of electronic products and websites more suitable for the elderly is also indispensable. For example, it is beneficial through the free adjustment of font and volume, encouraging the elderly to actively participate in the product design and testing process to make the product more user-friendly. Moreover, it is also an effective strategy to organize interest groups focused on digital skills and eHealth literacy in the community to promote communication and learning among the elderly people.

This study has certain limitations. Firstly, as the study was a cross-sectional survey, the causal relationship between social capital, eHealth literacy, and health behaviours could not be determined. In the future, a longitudinal study should be conducted. Secondly, only some elderly people from Jinan City were investigated. As individual social capital and eHealth literacy may vary between different countries and ages, we should pay attention to the extrapolation of the research results. Thirdly, the concept of social capital is complicated, and there is no unified and effective measurement tool. In addition, this study used eHEALS as a eHealth literacy measurement tool and did not analyse its internal structure. In the initial scale development, Norman and Skinner tested the eHEALS and concluded it is one-dimensional [28]. However, several studies found that it has two or even three dimensions [62, 63]. The uncertainties about the dimensions of the eHEALS can be a limitation. Therefore, future study should use different social capital assessment tools and consider the dimensions of eHealth literacy to verify and expand the study’s results. Finally, this study used self-report measures, which may have some information bias.

## Conclusions

This study demonstrates that social capital and structural social capital are related to eHealth literacy, and that eHealth literacy mediates the association between elderly people’s structural social capital and health behaviours. Therefore, social capital and eHealth literacy must be considered when designing and implementing health behaviour intervention programmes for elderly people.

## Data Availability

The Datasets can be made available to any interested person(s) contacting the corresponding author via email.

## Abbreviations

CSSCS: Chinese Shortened Social Capital Scale
eHEALS: eHealth Literacy Scale
HPLP: Health-Promoting Lifestyle Profile
